# Identification of MicroRNAs Linked to Regulators of Muscle Protein Synthesis and Regeneration in Young and Old Skeletal Muscle

**DOI:** 10.1371/journal.pone.0114009

**Published:** 2014-12-02

**Authors:** Evelyn Zacharewicz, Paul Della Gatta, John Reynolds, Andrew Garnham, Tamsyn Crowley, Aaron P. Russell, Séverine Lamon

**Affiliations:** 1 Centre for Physical Activity and Nutrition, School of Exercise and Nutrition Sciences, Deakin University, Burwood, Victoria, Australia; 2 Biostatistics Unit, Faculty of Health, Deakin University, Burwood, Victoria, Australia; 3 School of Medicine, Deakin University, Waurn Ponds, Victoria, Australia; 4 Australian Animal Health Laboratory, CSIRO Animal, Food and Health Sciences, Waurn Ponds, Victoria, Australia; University of Birmingham, United Kingdom

## Abstract

**Background:**

Over the course of ageing there is a natural and progressive loss of skeletal muscle mass. The onset and progression of age-related muscle wasting is associated with an attenuated activation of Akt-mTOR signalling and muscle protein synthesis in response to anabolic stimuli such as resistance exercise. MicroRNAs (miRNAs) are novel and important post-transcriptional regulators of numerous cellular processes. The role of miRNAs in the regulation of muscle protein synthesis following resistance exercise is poorly understood. This study investigated the changes in skeletal muscle miRNA expression following an acute bout of resistance exercise in young and old subjects with a focus on the miRNA species predicted to target Akt-mTOR signalling.

**Results:**

Ten young (24.2±0.9 years) and 10 old (66.6±1.1 years) males completed an acute resistance exercise bout known to maximise muscle protein synthesis, with muscle biopsies collected before and 2 hours after exercise. We screened the expression of 754 miRNAs in the muscle biopsies and found 26 miRNAs to be regulated with age, exercise or a combination of both factors. Nine of these miRNAs are highly predicted to regulate targets within the Akt-mTOR signalling pathway and 5 miRNAs have validated binding sites within the 3′ UTRs of several members of the Akt-mTOR signalling pathway. The miR-99/100 family of miRNAs notably emerged as potentially important regulators of skeletal muscle mass in young and old subjects.

**Conclusion:**

This study has identified several miRNAs that were regulated with age or with a single bout of resistance exercise. Some of these miRNAs were predicted to influence Akt-mTOR signalling, and therefore potentially skeletal muscle mass. These miRNAs should be considered as candidate targets for *in vivo* modulation.

## Introduction

Skeletal muscle serves as a structural and mechanical unit enabling the maintenance of posture and the performance of gross and fine motor movements. It is a highly plastic tissue, able to alter its size and metabolism to maintain optimal function in response to various exogenous and endogenous stimuli. Preserving skeletal muscle mass and health as we age is an essential component of whole body health and reduces the risk of chronic disease. However, by the age of 50 years, approximately 10% of muscle mass is lost and continues to decrease at an accelerated rate until death [1]. This age-related loss of muscle mass and the associated frailty syndrome is linked to a reduced capacity for muscle regeneration in aged rodents [2] and insufficient muscle protein synthesis (MPS) in elderly humans [3]. While regular physical activity and ingestion of good quality protein attenuates age-related wasting, they do not stop or reverse this process.

The Akt-mTOR signalling pathway is a major regulator of skeletal muscle mass via the positive and negative modulation of numerous downstream targets involved in the MPS process [4]. Resistance exercise is a potent stimulator of the Akt-mTOR pathway resulting in maximal MPS activation at an exercise intensity of 60% of 1 repetition maximum (1RM) 2 hours following the exercise bout. This activation is blunted but not delayed in elderly subjects [3]. So far, the molecular mechanisms underlying MPS are not fully understood.

MicroRNAs (miRNAs) are short single strands of nucleic acids (20–22 nt) that regulate numerous gene networks and signalling pathways [5], [6]. MiRNAs regulate gene expression by binding to messenger RNAs (mRNAs) and either directly degrading the mRNA or inhibiting protein translation [6], [7]. In rare cases, miRNAs can also stabilise mRNA targets [8]. MiRNAs are therefore essential post-transcriptional regulators in the cell. MiRNAs are implicated in the process of ageing [9], [10]. In mouse skeletal muscle, 57 miRNAs displayed aberrant expression in old mice when compared to young mice, with bioinformatics analysis predicting a role in the regulation of myogenesis for several of these miRNAs [11]. In humans, let-7b and let-7e, two miRNAs playing an anti-proliferative role in cancer cells [12], have a greater basal expression in the skeletal muscle of old subjects when compared to young subjects [13]. Thus far, two studies have investigated the association between miRNAs and exercise in an elderly population [14], [15]. MiR-1 expression was down regulated in young subjects but remained elevated in old subjects 3 and 6 hours following an acute bout of resistance exercise combined with the ingestion of essential amino acids (EAA) [14]. MiR-1 can inhibit IGF-1 [16], the latter a key stimulator of MPS [4]. Another study investigated a subset of 60 muscle enriched miRNAs in young and old subjects following an acute bout of resistance exercise. This study found 17 miRNAs that were downregulated 6 hours after an acute bout of resistance exercise in the young subjects only [15], including miR-126, a miRNA predicted to regulate IGF-1 signalling. Further analysis in C2C12 myotubes revealed that following IGF-1 treatment, miR-126 repression resulted in increased phosphorylation levels of various downstream targets of IGF-1 when compared to control cells treated with IGF-1. A sustained expression in miR-1 and miR-126 post-exercise may partly explain the reduced protein synthesis response observed in elderly subjects following resistance exercise. However, the role of miRNAs in the age-related loss of muscle mass requires further investigation.

The aim of this study was to perform a large-scale miRNA screening in skeletal muscle samples of young and old subjects following an acute bout of resistance exercise. Discriminant analysis, principal components analysis and bioinformatics analysis were performed on the miRNAs regulated by age, exercise or a combination of both. This allowed identification of miRNAs potentially involved in age-related muscle wasting with a specific focus on the Akt-mTOR pathway. Activation of the MPS pathways was indirectly assessed by measuring the expression levels of protein synthesis regulators.

## Materials and Methods

### Subjects

Subject characteristics have been previously described [17]. Briefly, 10 healthy young (18–30 years old) and 10 healthy older (60–75 years old) males gave their written consent to participate in the study. The subjects were not involved in any resistance training program and not taking any protein and anabolic supplementation. The study was approved by the Deakin University Human Research Committee (#2011–043). Power analysis conducted on the expected outcomes (α = 0.05, β = 0.2) indicated that a samples size of 20 (10 from each group) is sufficient to minimise the risk of a type II error. Two weeks prior to the trial a dual-energy x-ray absorptiometry scan (DXA) was performed on the subjects to assess their lean and fat mass composition as well as bone density. Subjects' 1RM was also determined.

### Experimental design

The experimental design has been previously described [17]. Briefly, following an overnight fast, a resting muscle biopsy was taken from a subject's vastus lateralis muscle at the Deakin University clinical trial facility. Immediately following the biopsy, subjects completed a leg extension exercise at 60% of their respective 1RM consisting of 3 sets of 14 repetitions with 2 min rest periods between each set. Two hours following the exercise a second biopsy was taken from the contralateral muscle.

### Protein extraction and western blotting

The protein extraction method has already been described [17]. Equal amount of protein was separated on either a 6% polyacrylamide self-cast gel as per previously published [17], a 4–12% NuPAGE Novex Bis-Tris Gel (Life Technologies, Mulgrave, VIC, Australia) or a 4–15% Criterion TGX Stain-Free Precast gel (Bio-Rad, Parkville, VIC, Australia) with the respective electrophoresis and transfer buffers provided by the suppliers. All proteins were transferred for 1.5 h onto Immobilin-FL PVDF membranes (Millipore, Billerica, MA, USA) with the exception of the phospho-mTOR blot which was transferred onto an Immun-Blot PVDF membrane (Bio-Rad, Parkville, VIC, Australia). The following proteins were probed: phospho-Akt^Ser473^ (9271), phospho-4E-BP1^Thr37/46^ (9451), phospho-p70 S6 Kinase^Thr389^ (9234), phospho-mTOR^Ser2448^ (5536), total Akt (9272), total mTOR (2972), total IGF-1R (3018), total RPTOR (2280) (Cell Signalling Technologies, Arundel, QLD, Australia); and GDF 8/11 (myostatin; 34781) (Santa Cruz, Scoresby, VIC, Australia). For western specifications see Table 1. Following overnight incubation the membranes were washed and incubated for 1 h with either a goat anti-rabbit IgG antibody labelled with an infrared-fluorescent 800 nm dye or a donkey anti-goat IgG with infrared-fluorescence 680 nm dye (Alexa Fluor 800, Life Technologies, Mulgrave, VIC, Australia) diluted in a buffer containing PBS and Odyssey blocking buffer (LI-COR Biosciences, Lincoln, NE, USA) at a 1∶1 ratio and 0.01% SDS, with the exception of the phospho-mTOR blots. After washing, the proteins were exposed on an Odyssey Infrared Imaging System (LI-COR Biosciences, Lincoln, NE, USA) and individual protein band optical densities were determined using the LI-COR Image Studio Lite software. The phospho-mTOR blots were probed with a HRP-labelled goat anti-rabbit IgG (Perkin Elmer, Glen Waverly, VIC, Australia) and detected by chemiluminescence (Clarity Western ECL Substrate, Bio-Rad Laboratories, Hercules, CA, USA) on a VersaDoc 4000 MP imaging system (Bio-Rad Laboratories, Hercules, CA, USA) with a 1 min exposure time. All blots were normalized to the GAPDH protein (G8795; Sigma-Aldrich, Sydney, NSW, Australia) with the exception of phospho-mTOR which was normalised to total protein load as determined by coommasie stain.

**Table 1:** Western blot antibody concentrations.

Primary antibody	Primary antibody dilution	Secondary antibody	Secondary antibody dilution	System used
phospho-Akt^Ser473^	1∶500	Goat anti-rabbit IgG	1∶5000	NuPAGE SDS-PAGE Gel System
phospho-4E-BP1^Thr37/46^	1∶500	Goat anti-rabbit IgG	1∶5000	NuPAGE SDS-PAGE Gel System
phospho-p70 S6 Kinase^Thr389^	1∶500	Goat anti-rabbit IgG	1∶5000	NuPAGE SDS-PAGE Gel System
phospho-mTOR^Ser2448^	1∶1000	Goat anti-rabbit IgG	1∶10000	SDS-PAGE
Total Akt	11000	Goat anti-rabbit IgG	1∶5000	Criterion TGX Stain-Free System
Total mTOR	1∶750	Goat anti-rabbit IgG	1∶5000	Criterion TGX Stain-Free System
Total IGF-1R	1∶500	Goat anti-rabbit IgG	1∶5000	NuPAGE SDS-PAGE Gel System
Total RPTOR	1∶1000	Goat anti-rabbit IgG	1∶5000	Criterion TGX Stain-Free System
Myostatin	1∶1000	Donkey anti-goat IgG	1∶5000	NuPAGE SDS-PAGE Gel System
GAPDH	1∶20000	Goat anti-mouse IgG	1∶5000	N/A

### RNA extraction and miRNA arrays

Total RNA was extracted from muscle biopsies (∼10 mg) using Tri-Reagent Solution (Ambion Inc., Austin, USA) according to the manufacturer's protocol. RNA (350 ng) was reverse transcribed using the Taqman microRNA Reverse Transcription (RT) kit and Megaplex RT Primers, Human Pool A v2.1 and Human Pool B v3.0 (Applied Biosystems, Life Technologies, Mulgrave, VIC, Australia). The RT reaction consisted of 2.7 mM dNTP, 0.3 U/µL RNase inhibitor, 3 mM MgCl_2_, 10 U/µL MultiScribe enzyme, 1× buffer and 1× primers. The RT conditions consisted of 40 cycles at 16°C for 2 min, 42°C for 1 min and 50°C for 2 min, followed by 5 min at 85°C to stop the reaction then cooled to 4°C. All PCR plates and films were purchased from Axygen Scientific, Inc, Union City, CA, USA.

MiRNA expression in the samples was assessed using the TaqMan Array Human MicroRNA A+B Cards v3.0 (Applied Biosystems, Life Technologies, Mulgrave, Australia). Collectively, these cards allow for the accurate quantitation of 754 human miRNAs including positive and negative controls. The results from the Megaplex were then analysed using ExpressionSuite Software v1.0 (Applied Biosystems, Life Technologies, Mulgrave, VIC, Australia) and the data was normalized using the global normalization function [18]. Ct values were then transformed into arbitrary units (AU).

### Bioinformatics analysis

Top cellular functions and miRNA-mRNA target interactions were determined using online software package Ingenuity System Interactive Pathway Systems (version 18488943). Stringency was set at ‘highly predicted’ and ‘experimentally validated’. Ingenuity pathway analysis (IPA) was used to generate figures depicting the relationship between miRNAs and predicted target mRNAs involved in MPS.

### Real-time PCR

RNA was treated with DNase I Amplification Grade (Invitrogen, Life Technologies, Mulgrave, Australia) and reverse transcribed using Oligo-dT primers and the High Capacity RNA-to-cDNA kit (Applied Biosystems, Life Technologies, Mulgrave, Australia) and then RNase H treated (Invitrogen, Life Technologies, Mulgrave, VIC, AUS) according to the manufacturer's instructions. Quantitative real-time PCR (Stratagene Mx3000, Stratagene La Jolla, CA, USA) was used to measure the expression levels of mTOR, IGF-1R, RPTOR and Foxo-1 genes. Sequences and conditions are described in Table 2. Expression was determined using Stratagene Mx3000 software and normalised to total single stranded cDNA content determined by Quant-it OliGreen ssDNA Assay Kit (Invitrogen, Life Technologies, Mulgrave, Australia).

**Table 2:** Primer sequences and conditions.

Target	Accession number		Primer sequence	Primer concentration (nM)	Probe sequence	Probe concentration (nM)
mTOR	NM_004958	**F**	CAAGAACTCGCTGATCCAAATG	100	TGCATTCCGACCTTCTGCCTTCA	50
		**R**	GCTGTACGTTCCTTCTCCTTC			
IGF-1R	NM_000875	**F**	AGTTATCTCCGGTCTCTGAGG	100	TCATCTTGCTCAGGCTTGGAGGTG	50
		**R**	TCTGTGGACGAACTTATTGGC			
RPTOR	AY_090663	**F**	TCGTCAAGTCCTTCAAGCAG	300	TCCTGCTCCCGCTGTAGTGC	150
		**R**	GGGTGATTTGGGTTGATTGC			
Foxo-1	NM_02015	**F**	AAGAGCGTGCCCTACTTCAA	300	N/A	N/A
		**R**	CTGTTGTTGTCCATGGATGC			

### Statistical methods

All data are reported as mean ±SEM. The mixed-model 2-way analysis of variance (ANOVA) was used to compare group means. Diagnostic plots of residuals and fitted values were checked to ensure homogeneity of variance (a key assumption for ANOVA). Consequently, all protein data was log_10_-transformed and analyses were conducted on these transformed scales. The least significant difference (LSD) test was used to compare pairs of means. The significance levels for both the F-tests in the ANOVA and the LSD tests were set at p<0.05.

#### Split plot ANOVA

AU values generated from the miRNA arrays were transformed by first adding a small constant (0.0001) and then taking the logarithm to base 2 of the result. The log transformation was required to uncouple a variance-mean relationship that was evident in preliminary analyses of variance of the AU values. A split-plot ANOVA with group (young or old) as the main-plot treatment and time (pre or post exercise) as the split-plot treatment was conducted on the log_2_ transformed AU values for each of the 754 miRNAs. First we estimated three contrasts and the p-values of their associated F-tests were recorded: (1) Main effect of old versus young, (2) main effect of post (exercise) versus pre (exercise), and (3) interaction contrast: (old, post) + (young, pre) – (old, pre) – (young, post). We then did pairwise comparisons and estimated their associated contrasts and p-values: (4) post versus pre in the young age group, (5) post versus pre in the old age group, (6) old versus young at the level of pre, and (7) old versus young at the level of post. In order to identify notable miRNAS with respect to these contrasts, the negative log_10_ transformation of the p-value was plotted against the associated contrast (on the log_2_ scale) in a “volcano” plot [19] for each of the 7 types of contrasts. Target miRNAs for which both the Young and Old age groups had means ≤1.219 on the log_2_ scale (Ct>32) were excluded from consideration. Target miRNAs with absolute values of their contrasts ≥1 and transformed p-values ≥1.301 (i.e. p≤0.05) were considered to be notable for that contrast. Analyses of variance were conducted using routines in the GenStat package [20] and graphs were produced in R version 2.15.1 (2012-06-22, Copyright 2012 The R Foundation for Statistical Computing).

#### Discriminant analysis

To search for an optimal set of miRNAs to discriminate between the two age groups, a stepwise discriminant analysis using forward selection based on Wilk's lambda was performed on a reduced set of the 26 statistically significant miRNAs identified in the ANOVAs. The optimal set was determined by choosing, from models with no more than 10 miRNAs, the model from the forward step with the minimum validation error. The misclassification rate of this selected model was estimated using 10-fold cross-validation. The DISCRIMINATE procedure in the GenStat package was used for this stepwise analysis.

#### Principal components analysis

A principal components analysis, using the PCP directive in GenStat, was used to identify linear combinations of the miRNAs, in the reduced set of 26, that appeared to account for most of the variation between individuals and to produce an ordination or “map” of the individuals based on the first two of the resulting principal components.

## Results

### Subject characteristics

The physiological characteristics of the subjects in this study have been previously published in Stefanetti et al [17]. Briefly, the average age of the two cohorts was 24.2±3.0 years old and 66.6±3.5 years old for the young and old group, respectively. No significant difference was observed in body mass, tissue composition and maximal voluntary contraction (1RM) between the two groups.

### MiRNA expression in young and old skeletal muscle following an acute bout of resistance exercise

**Figure 1:**
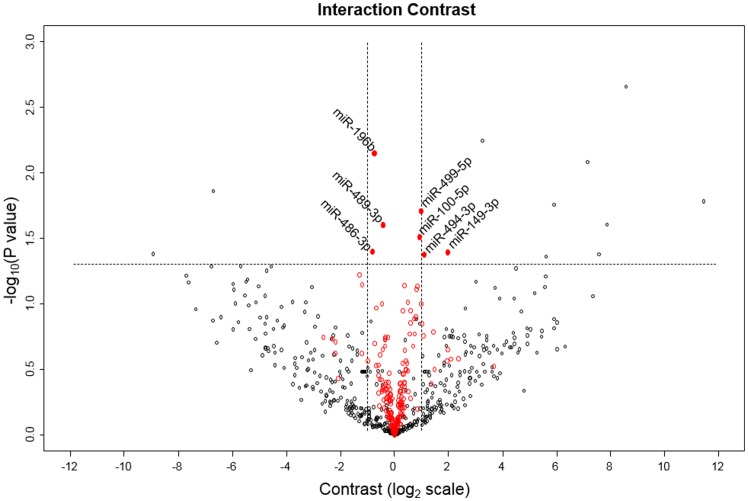
Volcano plot for the interaction contrast. This volcano plot depicts the interaction contrast and serves as a representative plot for the 7 contrasts estimated in the statistical analysis. The red points correspond to miRNAs with a Ct<32. The black points represent miRNAs with a Ct≥32. Statistical significance was set at p<0.05 corresponding to –log_10_(p value) = 1.3 on the y-axis and is represented by the dashed line plotted perpendicular to the y-axis. The dashed lines perpendicular to the x-axis represent a fold-change of 2 corresponding to log_2_(fold-change) = 1 on the x-axis.

**Figure 2:**
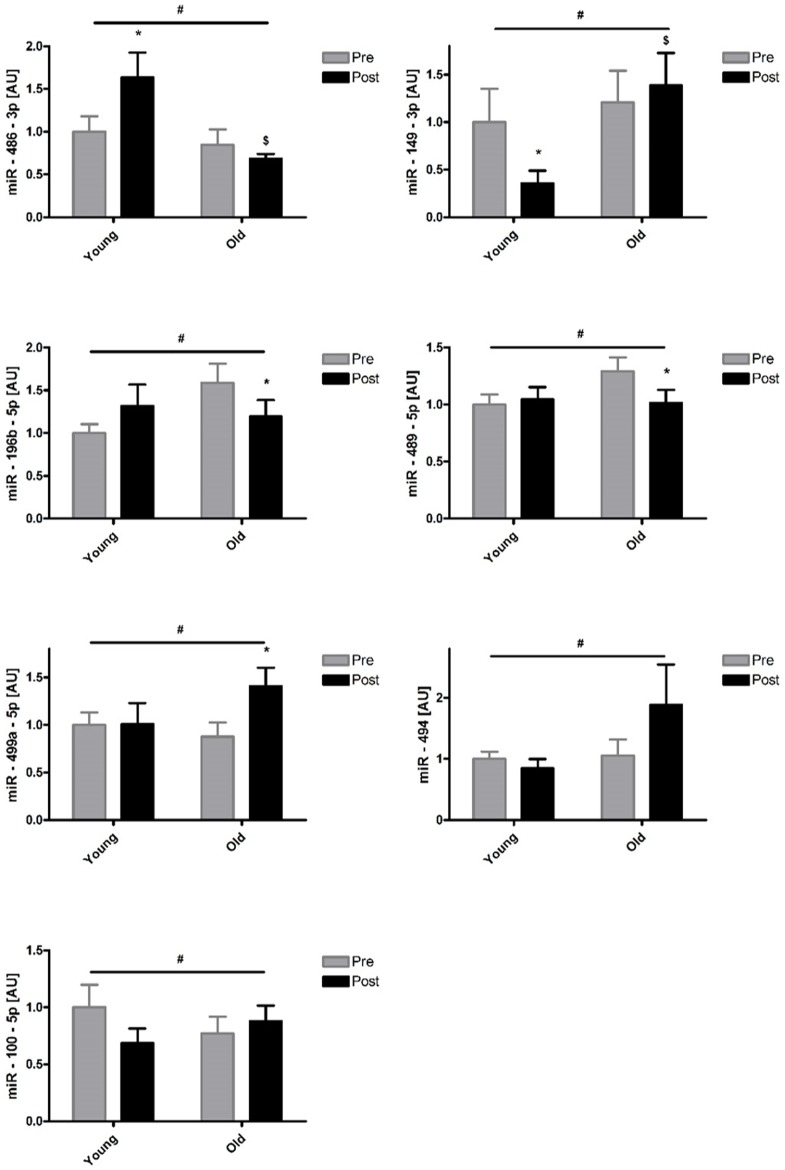
Expression levels of miRNAs with significant age by exercise interaction. Differences in miRNA level in young and old subjects 2 hours post exercise. * Significantly different from pre (p<0.05). $ Significantly different from young subjects at corresponding time point (p<0.05). The reported statistical significance is based on analysis of the transformed data but the reported means ±S.E.M. are on the original (untransformed) scale.

**Figure 3:**
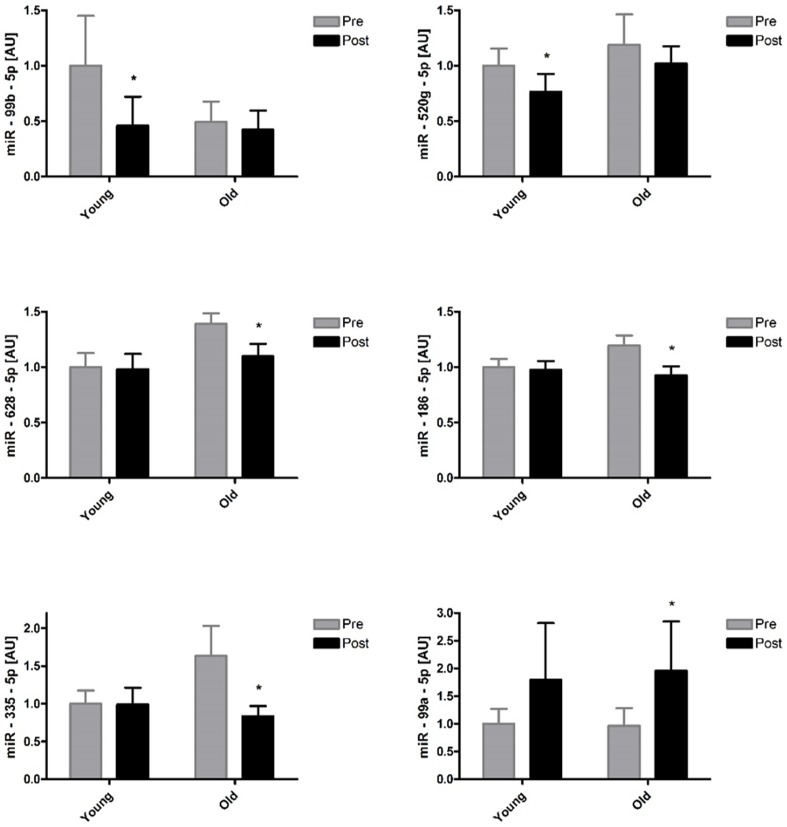
Expression levels of miRNAs with a significant exercise effect in one of the age groups but no interaction contrast. Differences in miRNA level in young and old subjects 2 hours post exercise. * Significantly different from pre (p<0.05). The reported statistical significance is based on analysis of the transformed data but the reported means ±S.E.M. are on the original (untransformed) scale.

The Taqman Array Human MicroRNA Cards Set 3.0 was used to assess the expression levels of 754 human miRNAs in the muscle samples. The relevant level of expression cut-off was set at Mean(Ct) <32 for each miRNA as recommended by the manufacturers. According to this criterion, 257 miRNAs (34%) were considered to be sufficiently expressed in skeletal muscle for further analysis. We investigated the statistical variation of all 257 miRNAs with age, exercise and a combination of both. A total of 7 contrasts were considered in the statistical analysis: (1) Main effect of age, (2) main effect of time, (3) interaction contrast: (old, post) + (young, pre) – (old, pre) – (young, post), (4) effect of time in the young age group, (5) effect of time in the old age group, (6) effect of age at the level of pre, and (7) effect of age at the level of post. A first selection based on a statistically significant p-value (p<0.05) and fold-change ≥2 for one or more of the contrasts yielded 7 miRNAs: miR-149-3p, miR-483-5p, miR-486-3p miR-494-3p, miR-500a-5p, miR-518b, and miR-99b-5p. Figure 1 depicts a volcano plot representing the interaction contrast. MiRNAs are plotted by fold change against p-value. Following this, a relaxed selection criterion that only considered a statistically significant p-value was applied. From this analysis, 26 miRNAs were returned for one or more of the contrasts. We observed a main effect of age and a main effect of exercise in 7 and 5 miRNAs, respectively, out of the 26 miRNAs (Table 3). Seven miRNAs had a significant age by exercise interaction, indicating that exercise had a differential effect on the expression of these miRNAs in young and old subjects (Figure 1). Further statistically significant differences between pairs of means (age or exercise) of these 7 miRNAs are depicted in Figure 2. An additional 7 miRNAs were identified to have a significant exercise effect in either the young or old age groups with no significant interaction contrast (Figure 3). Finally, 4 and 7 miRNAs were also differentially expressed with age at the level of pre and post respectively (Table 4).

**Table 3:** MiRNAs identified to be regulated with age and with exercise.

Main effect of age		Main effect of exercise	
MiRNAt	Old/young	MiRNA	Post/pre
miR-146a-5p	1.6	miR-151-5p	0.8
miR-191-5p	1.2	miR-339-3p	1.3
miR-320a	1.3	miR-483-5p	0.8
miR-483-5p	1.9	miR-574-3p	0.8
miR-486-3p	0.6	miR-935	0.8
miR-539-5p	1.8		
miR-628-5p	1.3		

Values are fold-change. MiRNAs are arranged alphanumerically.

**Table 4:** MiRNAs identified to be regulated with age at the levels of pre and post.

Old v young, pre		Old v young, post	
MiRNA	Old/young	MiRNA	Old/young
miR-191-5p	1.3	let-7b	1.4
miR-320a	1.4	miR-149-3p	3.9
miR-483-5p	2.0	miR-199a-3p	1.7
miR-628-5p	1.4	miR-483-5p	1.8
		miR486-3p	0.9
		miR-500a-5p	0.5
		miR-518b	0.5

Values are fold-change. MiRNAs are arranged alphanumerically.

### Classification of young and old subjects using a subset of miRNAs

#### Discriminant analysis

**Figure 4:**
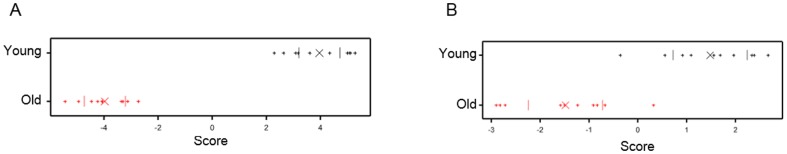
Graphical representation of the discriminant analysis. Two discriminant analysis models were used comprising of 9 miRNAs (A) and 4 miRNAs (B) selected with the smallest validation error. A. The first model correctly classified all subjects into their original age groups with old subjects (red) receiving negative scores and young subjects (black) receiving positive scores. B. The second model correctly classified 18 subjects into their original age groups and misclassified one old subject who received a positive score and one young subject who received a negative score. The mean for each group is represented by an X and standard deviation by vertical lines.

Discriminant analysis was used to check if a small set of linear combinations of the subset of 26 miRNAs could reproduce the classification of the subjects into age groups. The optimal model for classifying subjects into the two age groups required 9 of the 26 miRNAs (mir-196b-5p, miR-518b, miR-935, miR-99b-5p, miR-500a-5p, miR-494-3p, miR-335-5p, miR-520g-3p and let-7b) and the estimated misclassification rate was 0.2%. This particular model allocated all 20 subjects into their original age groups (Figure 4A). A second analysis was performed with a model restricted to the first 4 miRNAs (mir-196b-5p, miR-518b and miR-935 and miR-500a-5p) representing the most highly ranked components of the model. This particular model classified 18/20 subjects into their original age groups with an estimated error rate of 11.7%. One young subject and one old subject were misclassified (Figure 4B).

#### Principal Components Analysis

**Figure 5:**
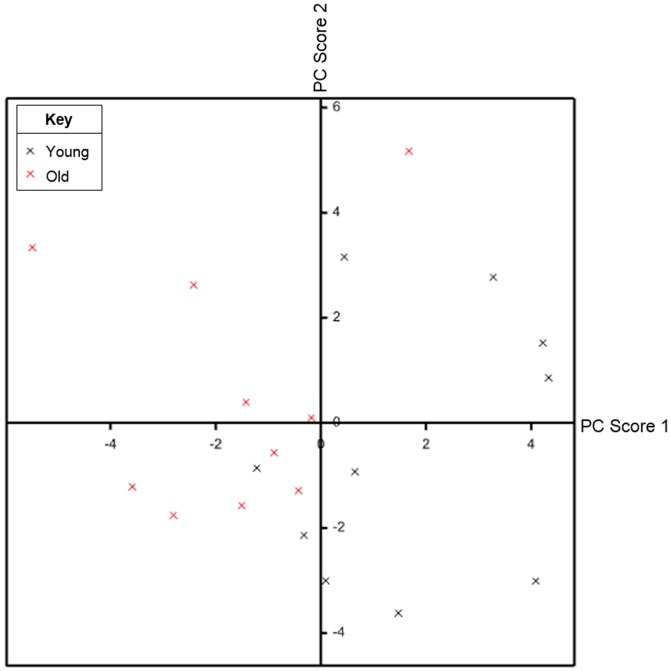
Graphical representation of the principal component analysis using the first two principal components. The first principal component was plotted on the x-axis and the second principal component was plotted on the y-axis. The first principal component separated the subjects by age group with young subjects (black) carrying mainly positive scores and old subjects (red) carrying mainly negative scores.

We investigated how much of the overall variation in the 26 miRNAs could be accounted for by a smaller number of dimensions or principal components. We found that 7 principal components accounted for 87% of the subject variation. The first principal component, which gave most weight to miR-518b, miR-335-5p, miR-500a-5p and miR-149-3p, separated the subjects into two distinct age groups with young subjects carrying mainly positive scores and old subjects carrying mainly negative scores (Figure 5). However, two young subjects carried negative scores and were therefore clustered with the old group and one old subject received a positive score. Of the young subjects, there are two distinct groups created from the second principal component, one in the upper right quadrant and one in the bottom half. On the other hand, most of the old subjects are clustered around the central and lower left quadrant but two outlying subjects are located in the upper left quadrant. The second principal component gave high weights to miR-539-5p, miR-518b, miR-99b-5p and miR-500a-5p.

### Bioinformatics analysis of miRNA functional role and specific target predictions

The bioinformatics analysis was performed on the miRNAs identified from 5 out of the 7 contrasts: (1) Main effect of age, (2) main effect of time, (3) interaction contrast: (old, post) + (young, pre) – (old, pre) – (young, post), (4) effect of time in the young age group, and (5) effect of time in the old age group. We used Ingenuity Systems Interactive Pathway Systems to identify cellular processes potentially being regulated by the miRNAs and predicted miRNA/mRNA interactions for the 23 miRNAs identified for the above-mentioned contrasts. Stringency criteria included that the putative interactions needed to be ‘highly predicted’ by the software algorithm or ‘experimentally observed’ in the literature. The miRNAs included in each analysis and the most highly ranked cellular functions likely to be regulated by each miRNA group are depicted in Table 5. The analysis revealed that 7 miRNAs within the 5 miRNA groups are predicted to regulate cellular growth and proliferation (p<0.05), namely miR-100-5p, miR-146a-5p, miR-196b-5p, miR-199a-3p, miR-489-3p, miR-99a-5p and miR-99b-5p.

**Table 5:** MiRNAs used in the bioinformatics analysis and top cellular processes potentially regulated by miRNAs.

MiRNA group	MiRNAs	Molecular and cellular function
Main effect of age	miR-146a-5p	Cellular function and maintenance
	miR-191-5p	Cellular development
	miR-320a	Cell death and survival
	miR-483-5p	
	miR-486-3p	
	miR-539-5p	
	miR-628-5p	
Main effect of exercise	miR-339-3p	Cellular assembly and organisation
	miR-483-5p	
	miR-515-5p	
	miR-574-3p	
	miR-935	
Interaction contrast	miR-100-5p	Cell cycle
	miR-149-3p	Cellular development
	miR-196b	Cellular growth and proliferation
	miR-486 3p	DNA replication, recombination and repair
	miR-489-3p	
	miR-494-3p	
	miR-499-5p	
Post versus pre in young subjects	miR-149-3p	Cell cycle
	miR-486-3p	Cellular development
	miR-518b	Cellular growth and proliferation
	miR-520g-3p	Cell death and survival
	miR-99b-5p	DNA replication, recombination and repair
Post versus pre in old subjects	miR-186-5p	Cellular development
	miR-196b	Cellular growth and proliferation
	miR-335-5p	Cell cycle,
	miR-499a-5p	DNA replication, recombination and repair
	miR-628-5p	
	miR-99a-5p	

**Figure 6:**
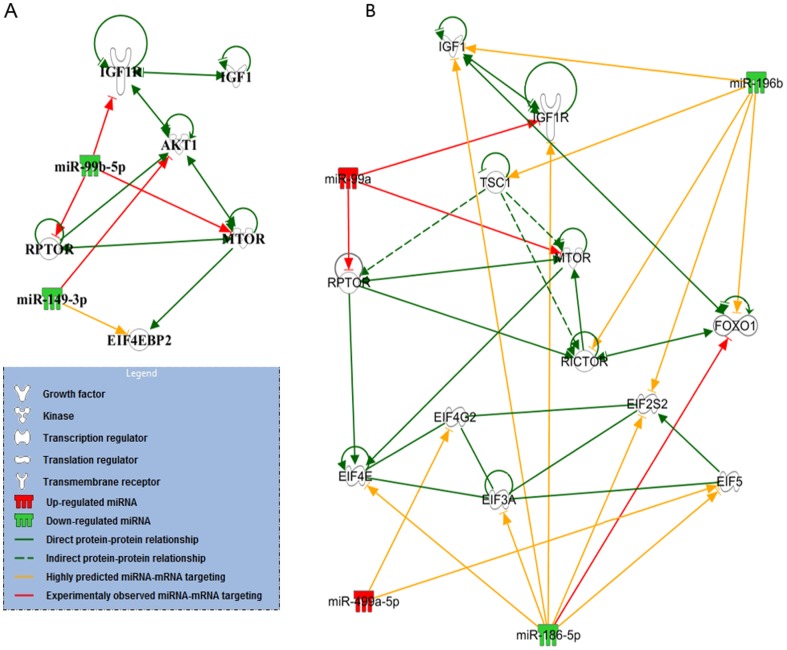
Ingenuity Pathway Analysis of miRNAs predicted to regulate muscle protein synthesis. MiRNAs predicted to target mRNAs within the muscle protein synthesis pathways in A. post versus pre in young subjects and B. post versus pre in old subjects. Gene targets included in figure are known regulators of muscle protein synthesis.

In addition, a list of predicted and validated mRNA targets for the 26 miRNAs included in the analysis was generated using Ingenuity software prediction algorithms. The genes known to regulate MPS were then selected for the pathway analysis, and figures were generated to illustrate the predicted regulatory differences in the MPS signalling cascades between young and old subjects (Figure 6). MiR-99a-5p, miR-99b-5p, miR-100-5p, miR-149-3p, miR-196b-5p and miR-199a all have validated gene targets within the MPS pathway (indicated by red arrows).

### Gene and protein levels of validated miRNA targets

**Figure 7:**
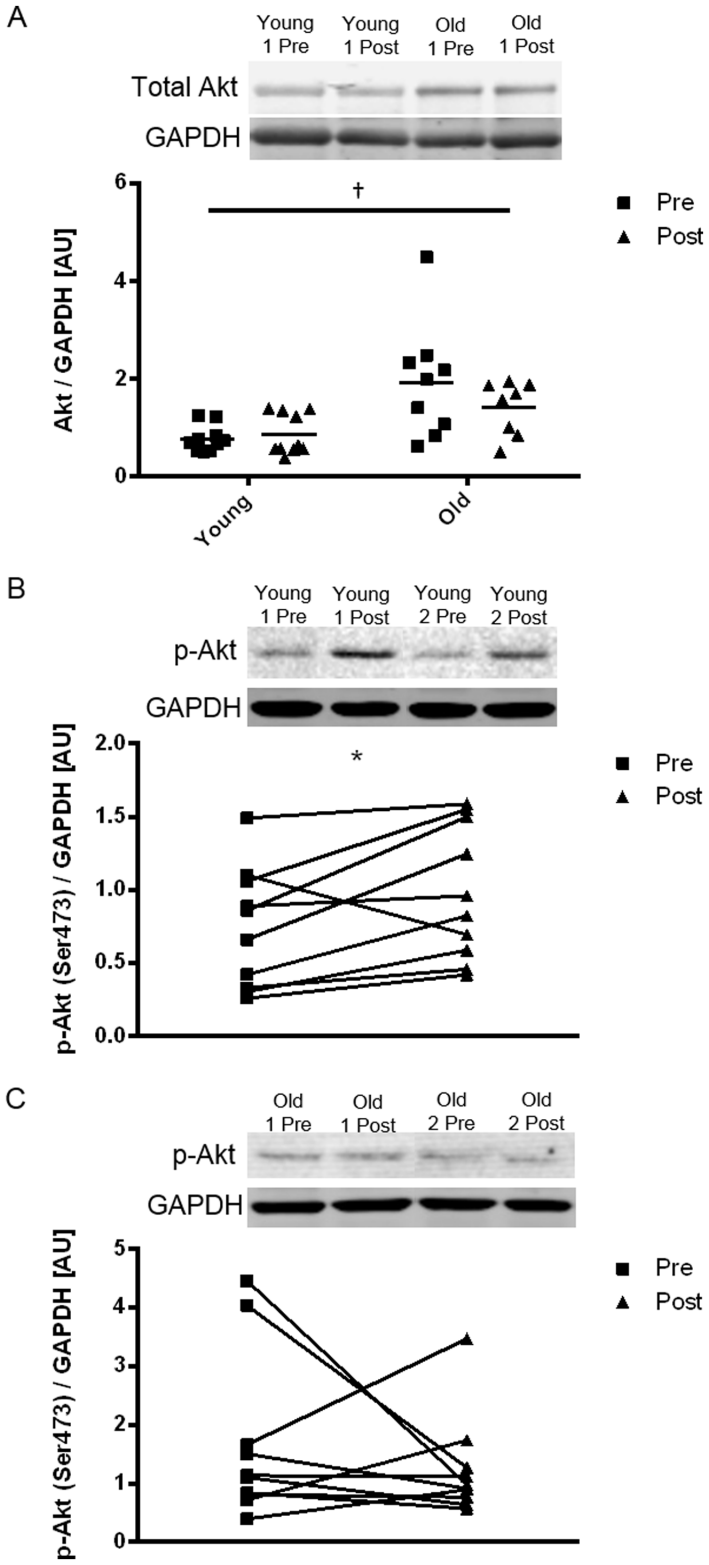
Expression levels of total Akt and phospho-Akt^Ser473^ protein in young and old subjects following exercise. Differences in total Akt levels in all subjects (A) and in phospho-Akt^Ser473^ levels in young (B) and old (C) subjects 2 h post exercise. Fold change is shown for individual subjects. †Significant age difference (p<0.05). *Significantly different from pre (p<0.05).

Following the bioinformatics analysis, the mRNA and proteins levels of mTOR, RPTOR, IGF-1R, Akt and Foxo-1 (previously published in [17]), which are validated targets of miR-99a-5p, miR-99b-5p, miR-100-5p, miR-149-3p, miR-196b-5p and miR-199a, were measured. Akt protein expression was elevated in the old subjects when compared to the young subjects (p<0.05) (Figure 7A). No significant effect of age and/or exercise was found for any of the remaining mRNA or proteins measured (See Figure S1).

### Validation of Akt-mTOR signalling pathway activation in young and old subjects following an acute bout of resistance exercise

Western blots were performed to determine if the members of the Akt-mTOR signalling pathway were activated through phosphorylation with the exercise bout. Two-way ANOVA showed no significant change in the expression levels of phospho-Akt, phospho-p70 S6 Kinase, phospho-4E-BP1 and phospho-mTOR with age and/or exercise. However, when looking at the different age groups, t-tests revealed an increase in Akt phosphorylation following exercise in the young subject group only (p<0.05) (Figure 7B and 7C).

## Discussion

Age-related muscle wasting is the natural and progressive loss of skeletal muscle mass and function that occurs with age. It results in severe impairments in strength, mobility, balance and endurance, and consequently, in decreased independence and quality of life in the elderly. Age-related muscle wasting is partially due to an attenuated activation of protein synthesis in response to resistance exercise [3]. The signalling proteins of the Akt-mTOR pathway, one of the major signalling pathways driving MPS [21], are aberrantly expressed in skeletal muscle of elderly subjects when compared to young subjects [3], [22]–[24]. However, the origin of this dysregulation is unknown. The aim of this study was to measure and compare the expression levels of miRNA species in young and old subjects at rest and following a resistance exercise protocol designed to maximise MPS. A secondary aim was to use statistics and bioinformatics analyses to identify miRNAs that may contribute to age-related muscle wasting by targeting members of the Akt-mTOR signalling pathway. This study identified 26 miRNAs that were regulated with age and/or exercise. Of these, 7 miRNAs were differentially regulated by age and exercise, and a further 7 miRNAs were regulated following exercise in either young or old subjects. Five of these 14 miRNAs have been identified as aberrantly expressed in several muscle disorders such as Duchenne muscular dystrophy and Myoshi myopathy [25]. Bioinformatics analysis predicted 7 of these miRNAs to regulate cellular growth and proliferation pathways. Specific mRNA target prediction also revealed that 9 of these miRNAs are potentially direct regulators of members of the Akt-mTOR signalling pathway. Six of these miRNAs, miR-99a-5p, miR-99b-5p, miR-100-5p, miR-149-3p, miR-196b-5p and miR-199a, have been previously validated to target several mRNAs that encode proteins within the protein synthesis pathways in different types of cells [26]–[32]. Of particular interest, miR-99a-5p, miR-99b-5p and miR-100-5p, members of the miR-99/100 family, share the same seed region and have validated target sequences within the 3′UTRs of mTOR, RPTOR and IGF-1R [26], [29], [30], [32]. In addition miR-199a targets the 3′ UTR of IGF-1 and mTOR [27], [33], while miR-149-3p and miR-196b-5p target the 3′ UTR of Akt1 [31] and Foxo-1 [28], respectively. These studies also demonstrated concomitant decreases or increases in the protein, and in some cases the mRNA, levels of the respective targets following the transfection of miRNA mimics or inhibitors, respectively. The mode of inhibition of miRNAs is largely dependent on its binding capacity to the 3′ UTR [34]. Interestingly, transfection of miR-99a-5p in PHK and HaCaT cells results in a decrease in IGF-1R protein but not mRNA expression, while transfection of miR-99b-5p and miR-100-5p into HaCaT cells decreased IGF-1R at both the mRNA and protein level. All three miRNAs share a common seed region and therefore a common binding site within the IGF-1R 3′ UTR and presumably inhibit the target in the same way. We report a greater basal amount of total Akt protein in the older cohort when compared to the young cohort. Elevated Akt expression in old skeletal muscle is not without precedent in the literature [24]. Akt is a direct target of miR-149-3p, which was significantly downregulated with exercise in the young subjects only. No correlation could be made between miR-149-3p expression and Akt expression and we did not report any change in the mRNA and protein expression levels of mTOR, RPTOR, IGF-1R and Foxo-1. A similar case has previously been reported where differences in miRNA expression in healthy and unhealthy muscle tissue were not paralleled by changes in the expression of predicted protein targets [35]. MiRNA regulation of mRNA targets is time-dependent [36] and may explain why no change was observed in mRNA and protein expression 2 hours following an acute exercise bout. It is possible that changes in these miRNAs may result in the regulation of their targets at a later time point post exercise, however such a temporal response requires further investigation. Interestingly, the individual expression patterns of miR-99a-5p, miR-99b-5p and miR-100-5p demonstrate some inconsistencies, and this suggests an added degree of complexity with respect to the mode of activity of miRNAs. Based on the currently understood principles of target binding, it could be expected that the function of these three miRNAs overlap [34]. However, *in vitro* studies have demonstrated that manipulating the expression of these miRNAs individually is sufficient to induce changes in target mRNA and/or protein expression without a compensatory/rescuing effect of the other miRNAs [26], [29], [30], [32]. How the overall expression of the miR-99/100 family of miRNAs affects target mRNA/protein expression in this study is not clear. The differential regulation within and between miRNAs sharing a common seed region suggests a multifaceted mode of regulation of mRNA targets.

Muscle enriched miRNAs (myomiRs), miR-1, miR-133a, miR-133b, miR-206, miR-486-5p and miR-499a-5p were amongst the most highly expressed miRNAs within the muscle of this cohort. With the exception of miR-499a-5p, the expression of these miRNAs was not altered with exercise or age. One study has investigated the expression of the myomiRs miR-1, miR-133a and miR-206 following an acute bout of resistance exercise in the skeletal muscle of young and old subjects. Drummond et al. reported a decrease in miR-1 expression with a concomitant increase in protein synthesis 3 and 6 hours post exercise and ingestion of an essential amino acid (EAA) solution in young subjects only [14]. This study also reported a delayed increase in protein synthesis in old subjects 6 hours post exercise with no change in miR-1 expression [14], [22]. A regulatory feedback process exists between miR-1 and IGF-1, an upstream regulator of Akt-mTOR, where miR-1 can directly target and inhibit IGF-1 and IGF-1 signalling can inhibit miR-1 expression [16]. Our study demonstrates that miR-1 was not regulated by resistance exercise alone 2 hours post exercise. The regulation of miR-1 may be subject to EAA availability during MPS as it is also responsive to EAA ingestion alone [37]. The combined effects of resistance exercise and EAA ingestion may also be required for the regulation of other myomiRs, but this is yet to be investigated.

Another study investigated the change in 60 muscle miRNA expression following an acute bout of resistance exercise [15]. This study found 17 miRNAs that were reduced 6 hours after an acute resistance exercise bout in young subjects only. In contrast we found 13 miRNAs that were either upregulated or downregulated in both young and old subjects 2 hours after acute exercise. Members of the Akt-mTOR signalling pathway were predicted targets for both sets of miRNAs. Overall, these studies suggest a dynamic regulation of miRNAs in skeletal muscle following resistance exercise and reveal the important role of miRNAs in the regulation Akt-mTOR signalling. Determining how these miRNAs might impact on the adaptive response of skeletal muscle to exercise will be essential in understanding the mechanisms of skeletal muscle growth and age-related muscle wasting.

MiR-486-3p originates from the opposite arm of miR-486-5p, a recognised myomiR, and was differentially regulated with age and exercise. MiR-486-3p expression was downregulated in old subjects when compared to young subjects and upregulated in young subjects only following exercise. MiR-486-3p has predicted targets within the MPS pathway, including several eukaryotic initiation factors (eIFs), RPTOR and TSC-1. The miR-486 stem-loop sequence is embedded within the Ankyrin-1 gene and is positively regulated by MRTF-A and SRF [38], two important regulators of muscle function. The differential regulation of miR-486-3p in young and old subjects may reflect an attenuated activity of the MRTF-A/SRF axis with age [39]. In contrast, no change was observed for miR-486-5p expression, suggesting that miR-486-3p and miR-486-5p expression are regulated independently of each other at the post-transcriptional level. This is not without precedent, as differential expression of other sense and antisense miRNA pairs has been described when comparing metastatic and non-metastatic prostate cancer tissue [40].

MiR-199a-3p and miR-199a-5p provide another example of differential expression of sense and anti-sense miRNA pairs. We observed a significantly higher miR-199a-3p (p<0.05) expression in old subjects when compared to young subjects in the post exercise biopsies. On the other hand, miR-199a-5p was either minimally expressed or not expressed in both young and old subjects. The expression of miR-199a-5p in healthy adult skeletal muscle has been reported as low relative to disease tissue [25]. Two stem-loop sequences exist for miR-199a, which originate from the dynamin 2 and dynamin 3 genes. MiR-199a-3p is upregulated during differentiation of myoblasts isolated from patients with Duchene Muscular Dystrophy (DMD) [41]. Overexpression of miR-199a-3p in C2C12 myoblasts inhibits differentiation and is associated with a decrease in Myf5, MyoD, myogenin and myosin heavy chain (MHC) [33]. On the other hand, inhibition of miR-199a-3p expression results in an increase in both myogenin and MHC as well as myotube hypertrophy [33]. This occurred with a concomitant increase in the protein levels of its validated targets IGF-1 and mTOR and a decrease in MuRF-1 protein; the latter a regulator of protein degradation [42]. We have previously reported a significant increase in MuRF-1 mRNA, but not protein, in the muscle samples from the subjects in the present study [17]. The increase in miR-199a-3p in old subjects may therefore be associated to an increase in MuRF-1 mRNA expression in older subjects.

The loss of skeletal muscle mass in the elderly occurs in parallel with numerous impairments, including a loss of regenerative capacity [43] and a reduced ability to maintain/replenish adenosine triphosphate (ATP) levels during exercise [44]. In addition to the miRNAs regulated by resistance exercise, we identified several miRNAs displaying differential expression levels at rest in young and old subjects. Understanding the potential role of miRNAs in the onset and progression of age-related muscle dysfunction is of major interest. The let-7 family of miRNAs is associated with ageing in *Caenorhabditis elegans* and humans [10], [13]. At rest, let-7b was upregulated in old subjects when compared to young subjects. Drummond et al. previously reported an upregulation in the expression levels of let-7b and let-7e, both belonging to the let-7 family of miRNAs, in skeletal muscle biopsy samples of old subjects [13]. Expression levels of let-7b are associated with a negative regulation of the cell cycle. Drummond et al. demonstrated a concomitant decrease in the cell cycle regulators CDK6, CDC25A and CDC34 in old subjects, which might provide an explanation for reduced regeneration capacity. On the other hand Rivas et al. found a decrease in let-7f expression in elderly skeletal muscle and no difference in any other let-7 miRNAs [15]. More research is required to elucidate the role of let-7 miRNAs in the reduced regenerative capacity of aged skeletal muscle.

MiR-320a is another miRNA that was elevated in the old subjects. MiR-320a activity is linked to glycolysis by directly downregulating phosphofructokinase, the rate limiting glycolytic enzyme [45]. MiR-320a expression is decreased in tissues and cells characterised by increased glycolysis, including lung cancer tissue, disused diaphragm and C2C12 myotubes subjected to oxidative stress [45]. While an impairment in glycolysis has been demonstrated in elderly subjects [44], the reasons for this dysregulation remain unclear. By negatively regulating a critical enzyme of the glycolysis pathway, elevated endogenous levels of miR-320a may therefore account for a decreased capacity of the glycolytic system in the elderly.

In this study we sought to determine if there were any impairments in the Akt-mTOR signalling pathway in the old subject group when compared to the young subjects group. With regards to the expression levels of the molecular markers of protein synthesis, we did not observe any differences in the phosphorylation levels of the selected proteins with exercise and/or age; with the exception of phospho-Akt, that was elevated in most of the subjects post exercise (figure 7B and 7C), but reached statistical significance when considering the young subject cohort only. It is possible that the phosphorylation levels of these proteins may have returned to baseline by 2 hours post exercise as observed previously by others [3], [46]. It must also be acknowledged that the old cohort may not be representative of the average elderly population in terms of physical activity levels and fitness levels; a problem faced by many exercise-based trials. Whilst this study only sought to investigate the expression levels of miRNAs in skeletal muscle 2 hours following exercise, when MPS is maximised in the subjects [3], a time course analysis of miRNA expression following resistance exercise may be more informative as to the degree of regulation of Akt-mTOR signalling by miRNAs. We also observed elevated total Akt levels in the old subjects, a finding previously observed by our group. Elevated levels of total Akt protein may be indicative of a reduced efficiency of the old subjects to phosphorylate the available Akt pool following the resistance exercise bout [24]. Finally, we found a tendency (p<0.1) for the myostatin protein, an inhibitor of Akt signalling and therefore a negative regulator of muscle mass, to be elevated in the muscle from the older cohort (data not shown) when compared to the younger subjects; an observation reported previously [24], [47].

The purpose of the discriminant analysis in the present study was to categorically group subjects into a young and old age group based on miRNA expression. A discriminant analysis with miR-196b-5p, miR-518b, miR-935 and miR-500a-5p misclassified one young and one old subject into the opposite age group. No previous studies have investigated the role of these miRNAs in skeletal muscle or age. MiR-196b-5p had a significant interaction contrast and was found to be down regulated post exercise in the old subjects. MiR-518b was upregulated in young subjects following exercise while miR-935 was significantly downregulated post exercise in all subjects. Finally, miR-500a-5p was found to be upregulated in old subjects when compared to young subjects. Only miR-196b-5p had predicted targets within the Akt-mTOR pathway. Notably, miR-196b-5p downregulation has been linked to an upregulation in TGF-β signalling in cutaneous systemic sclerosis . MiR-196b-5p regulates type I and II collagens, which are involved in the enrichment of the extracellular matrix (ECM) [49]. Elevated TGF-β signalling and ECM enrichment has been previously reported in elderly subjects and has been linked to the reduced regenerative capacity of muscle satellite cells [50], [51]. However, the link between miR-196b-5p and satellite cell regeneration is yet to be investigated.

## Conclusion

This study identified 26 miRNAs that were differentially regulated with age and/or exercise following an acute resistance exercise bout completed by young and old subjects. A subset of these miRNAs were predicted or have been previously associated with MPS and muscle regeneration. Further functional investigations that are beyond the scope of this study are required to determine the nature of the interactions between these miRNAs of interest and their targets. In addition, a time course pattern of miRNA and mRNA expression analysis may provide more clarity on the role and regulation of miRNAs in response to exercise in young and older individuals.

## Supporting Information

Figure S1 **Western blot bands of proteins directly targeted by identified miRNAs as assessed by IPA.** Western blot bands for total Akt (A), total mTOR (B), total RPTOR (C) and total IGF-1R (D) and the corresponding GAPDH loading control.
(PDF)
