# Supplementary figures and images for: Identification of MicroRNAs Linked to Regulators of Muscle Protein Synthesis and Regeneration in Young and Old Skeletal Muscle
Source: PLoS One. 2014 Dec 2;9(12):e114009. doi: 10.1371/journal.pone.0114009 (PMC4252069; doi:10.1371/journal.pone.0114009)

A

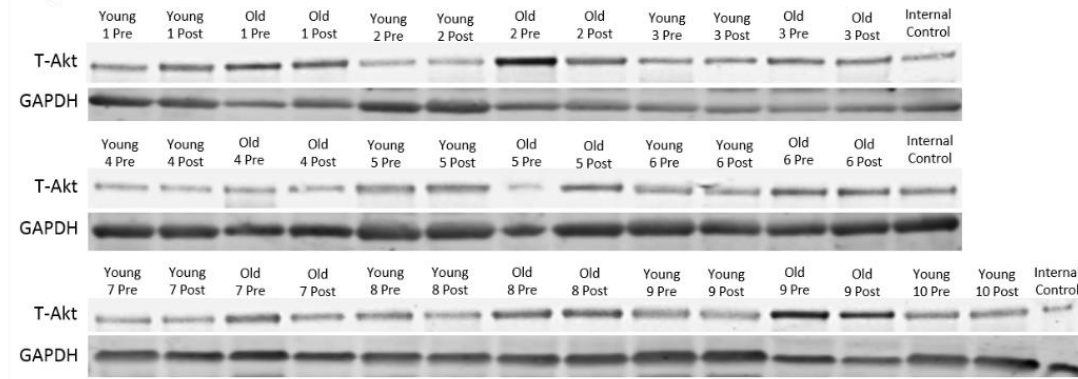

C

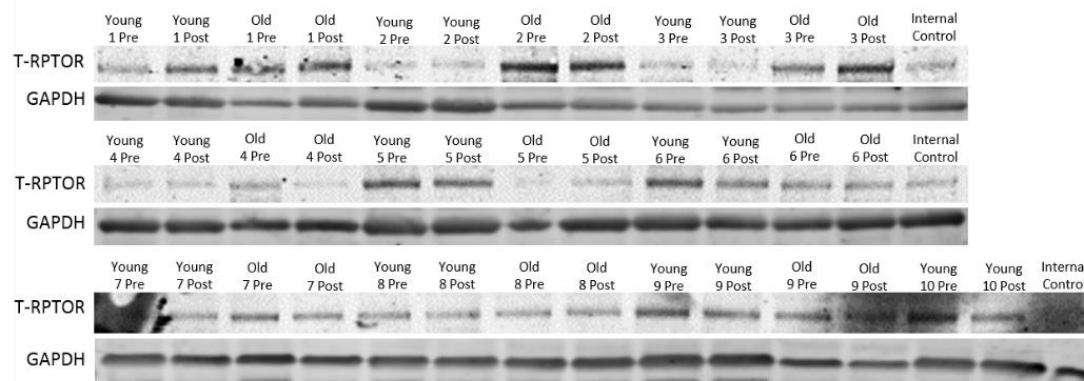

B

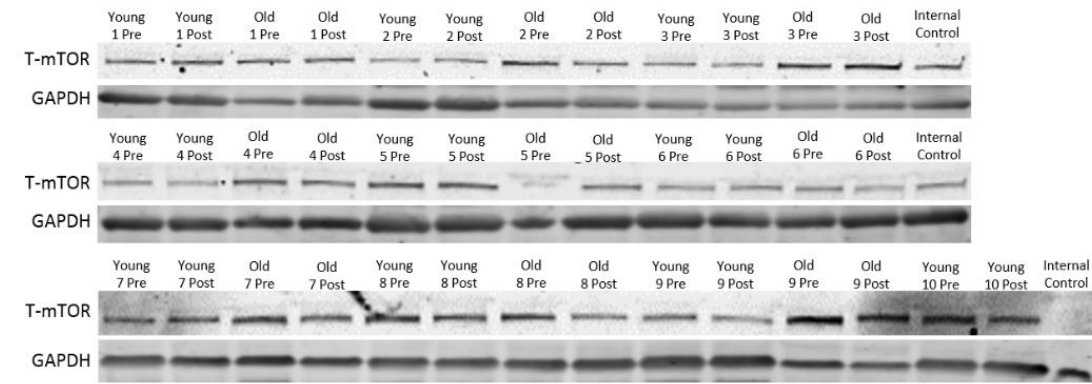

D

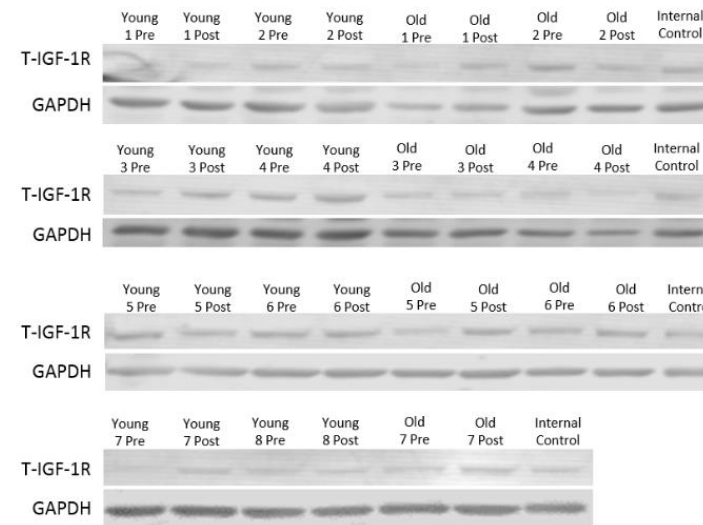

Supplement: Figure S1 — Western blot bands of proteins directly targeted by identified miRNAs as assessed by IPA. Western blot bands for total Akt (A), total mTOR (B), total RPTOR (C) and total IGF-1R (D) and the corresponding GAPDH loading control. (PDF) [file pone.0114009.s001.pdf]
